# Clinical and Genetic Description of Hereditary Chronic Pancreatitis in Pakistani Children

**DOI:** 10.5152/tjg.2023.22791

**Published:** 2023-10-01

**Authors:** Huma Arshad Cheema, Zafar Fayyaz, Anjum Saeed, Muhammad Nadeem Anjum, Sadaqat Ijaz, Muhammad Arshad Alvi, Syeda Sara Batool

**Affiliations:** 1 Division of Pediatric Medicine, Department of Pediatric Gastroenterology, Hepatology & Nutrition, The Children’s Hospital Lahore, University of Child Health Sciences, Lahore, Pakistan; 2 Department of Forensic Sciences, University of Health Sciences, Lahore, Pakistan

**Keywords:** Hereditary pancreatitis, trans-heterozygotes, PRSS1, SPINK1, Pakistan

## Abstract

**Background/Aims:**

The purpose of this study was to identify the spectrum and frequency of pathogenic variants as well as the clinical and genetic insight of hereditary chronic pancreatitis in Pakistani children.

**Materials and Methods:**

The deoxyribonucleic acid of affected probands of 44 unrelated Pakistani families, having hereditary chronic pancreatitis-affected children, were subjected to massive parallel sequencing for candidate reported genes (SPINK1, PRSS1, CFTR, CPA1, CTRC, CBS, AGL, PHKB, and LPL). Data were analyzed using different bioinformatics tools for the variants and in-silico analysis. All the identified variants were validated by direct sequencing of the targeted exons in the probands and their parents.

**Results:**

There were 50 patients included in this study with confirmed hereditary chronic pancreatitis. Nine known mutations in SPINK1, PRSS1, CFTR, CTRC, CBS, and AGL genes, and 10 novel variants in LPL, CFTR, CTR, and PHKB genes were identified. The identified variants were found in heterozygous, compound heterozygous, and trans-heterozygous forms, with rare allele frequency in the normal population. The novel variants were [c.378C>T(p.Lys126Asn) and c.719G>A(p.Arg240Gln) in CTRC, c.586-3C>A and c.763A>G(p.Arg255Gly) in CPA1, c.1160_1161insT(p.Lys387Asnfs*26), c.784C>T(p.Gln262*), c.1139+1G>A, c.175G>A(p.Gly59Arg) in LPL, c.388C>G(p.leu130val) in CFTR, and c.2327G>A(p.Arg776His in PHKB)]. The phenotypic characteristics were variable and correlated with the relevant variant.

**Conclusions:**

The genetic composition plays a significant role in the predisposition of hereditary chronic pancreatitis. The clinical presentation varies with the genetic determinant involved. This information would help in building up a diagnostic algorithm for our population that can be used for genetic screening services in affected cohorts.

**Main Points**

- Hereditary pancreatitis is an important and most common etiological factor of chronic and recurrent pancreatitis in children.
- Advances in genetics have led to a better understanding of the molecular basis of pancreatitis and the management of these patients.
- A variety of causative gene mutations are now known; novel mutations continue to be discovered.
- The first ever largest description of genetic and clinical aspects of hereditary pancreatitis in Pakistani children from the highly inbred community with significant novel variants is presented from a single tertiary care center.
- Genotype–phenotype correlations are difficult to determine and multi-center collaboration will likely be the need of the hour to characterize the clinical features with underlying monogenic disorders for pancreatitis.

## Introduction

Hereditary pancreatitis is one of the frequently described causes of recurrent acute and chronic pancreatitis in children in addition to hepatobiliary diseases. The incidence of hereditary pancreatitis among chronic pancreatitis cases is 0.3 to 0.5/100 000.^1,2^ There is no gender bias, but racial preponderance does exist, and it is more frequently reported in black ethnic origin in the West.^2^

The clinical presentation of hereditary chronic pancreatitis (HCP) is not different from the chronic pancreatitis of other etiologies. Abdominal pain with or without vomiting is the major clinical presentation followed by failure to thrive, steatorrhea, and family inheritance.^3,4^ Complications are part of chronic pancreatitis and invariably happened in the affected children, but with appropriate management of their various clinical manifestations, most of them can have a good quality of life.^5^

Most of the cases of recurrent acute pancreatitis and chronic pancreatitis in the past were considered idiopathic, but with the recent advances in genetic and autoimmune studies, the scenario has changed. Biochemical parameters, including serum amylase and lipase, are not helpful in these children owing to the chronic nature of the disease and severe atrophy of pancreatic tissue. Radiological modalities are most valuable in delineating the structural changes in HCP.^6^

Trypsin is activated intestinally by serine protease enteropeptidase while its auto-activation (trypsin-mediated trypsinogen) occurs in the pancreas. Mutations in cationic trypsinogen that increase auto-activation are strong risk factors for chronic pancreatitis, typically associated with hereditary pancreatitis. Protective mechanisms that cut down trypsinogen activation in the pancreas are either trypsin inhibition or trypsinogen degradation. Thus, pancreatitis is caused by uncontrolled trypsin activity either by increasing the trypsinogen activation to trypsin or by impairing the protective trypsinogen degradation and/or trypsin inhibition.^7^

The genetic analysis for the diagnosis of HCP is a sensitive and powerful tool. Different genetic mutations have been described in the literature with variable presentation and the majority of them have an autosomal dominant pattern of inheritance. The common genes involved in HCP are *PRSS1* (prevent proper auto inactivation of enzyme), *SPINK1*, *CTRC* (regulate trypsin inhibition and inactivation), *CFTR* (clear trypsin from the Pancreas), *CPA1*, and several other trypsin-dependent genetic causes.^1,2,8^ The clinical heterogeneity of pancreatic disease is at least partly due to allelic heterogeneity. Irrespective of the advancement in molecular biology, the underlying genetic cause of almost 30% of the affected families having hereditary pancreatitis is still unknown. The pathogenic variants of hereditary pancreatitis have not been evaluated in Pakistani children, so we aimed to identify the spectrum and frequency of pathogenic variants as well as the clinical and genetic insight into HCP in Pakistani children. 

## Materials and Methods

### Patients Recruitment

This is a cross-sectional, descriptive retrospective study of children having chronic pancreatitis with the demonstration of genetic variants. The study was conducted at the Department of Pediatric Gastroenterology, Hepatology and Nutrition from January 2017 to December 2020. During this period, 78 Pakistani children clinically diagnosed with chronic pancreatitis were enrolled for genetic analysis. This study was approved by the institutional review board of Children’s Hospital and University of Child Health Sciences, Lahore Pakistan (Approval No: 2019-22-CHICH) and conducted according to the principles of the Helsinki Declaration. Informed written consent from parents /guardians was obtained prior to their enrollment in the study.

The inclusion criteria were adopted from INSPPIRE definition of chronic pancreatitis.^8^

### INSPPIRE Definition of Chronic Pancreatitis

It requires at least 1 of the following:

1. Abdominal pain consistent with pancreatic origin with imaging findings suggestive of chronic pancreatitis.
2. Evidence of exocrine pancreatic insufficiency with imaging findings suggestive of chronic pancreatitis.
3. Evidence of endocrine insufficiency with imaging findings suggestive of chronic pancreatitis.
4. Surgical or pancreatic biopsy specimens demonstrating histopathologic features compatible with chronic pancreatic (CP).

### Radiological Modalities

Imaging examination including ultrasound abdomen, computed tomography (CT), magnetic resonance imaging, and magnetic resonance cholangiopancreatography (MRCP) was performed to identify the findings suggestive of chronic pancreatitis and included pancreatic parenchymal changes like heterogeneity, pancreatic calcifications, dilatation or irregularity of main pancreatic duct, or pancreatic atrophy. 

### Genetic Testing

Genomic deoxyribonucleic acid (DNA) was extracted from ethylenediamine tetraacetic acid (EDTA)-treated peripheral blood samples by using a QIAamp DNA Blood Mini kit (Qiagen, Hilden, Germany). Next-generation sequencing (NGS) was performed at CENTOGENE (Germany). Briefly, the extracted DNA was enzymatically fragmented and regions of interest were selectively enriched using capture probes and targeted against coding regions of ~6700 genes with known clinical significance. Libraries were generated with Illumina-compatible adaptors and sequenced on an Illumina platform. The evaluation was focused on coding exons along with flanking ±10 intronic bases within the captured region. Variants identified by NGS were Sanger sequenced to exclude any artifacts. Two independent copy number variation (CNV) callers were used to determine CNVs within the panel genes from the NGS data. All clinically relevant CNVs were confirmed with an orthogonal multiplex ligation-dependent probe amplification (MLPA) method. 

### Statistical Analysis

Statistical analysis was carried out by using the Statistical Package for Social Sciences version 23 (IBM Corp.; Armonk, NY, USA). Frequencies and percentages were calculated for the different qualitative variables, while mean and standard deviation were calculated for quantitative variables. Alignment of reference sequences was performed using “bl2seq” (www.blast.ncbi.nlm.nih.gov/), an online tool to localize the variant DNA sites Mutation Taster (www.mutationtaster.org)and PolyPhen-2 (http://genetics.bwh.harvard.edu/pph2/) was employed to predict the pathogenicity of the identified genomic variants. Human Splice Finder, Berkeley Drosophila Genome Project, Splice Port, and Net-Gene2 server were used to predict the pathogen city of splicing mutation. Crystal structure, protein stability, and simulation were computed using “PyMol” (https://pymol.org/2/), “CUPSAT” (http://cupsat.tu-bs.de/index.jsp), and “SDM” (http://marid.bioc.cam.ac.uk/sdm2/prediction).

## Results

### Patients and Clinical Outcomes

Seventy-eight children were initially recruited as having pancreatitis and enrolled during 4 years study period. Out of these 78 children, the molecular genetic diagnosis of 50 children (64%) was consistent with their clinical diagnosis. The remaining 28 children (36%) were clinically categorized as “unknown with/without complications.” Among these 50 children that were genetically positive for pancreatitis, 31 (62%) were females with the mean age at presentation ranging from 3 to 18 years (mean ± SD: 6.81 ± 2.3) as shown in Table 1. Thirty-four children (68%) had consanguinity and 25 children (50%) had a positive family history of pancreatitis (Supplementary Table 1). The most common presentation in our cohort was abdominal pain in 49 (98%) and vomiting in 45 (90%) children. The next most commonly reported symptoms were failure to thrive in 34 (68%), fever in 19 (38%), ascites in 7 (14%), jaundice in 4 (8%), steatorrhea in 3 (6%), and diabetes in 3 (6%). Diagnostic modalities included a high index of clinical suspicion of chronic pancreatitis and radiological modalities including CT and MRCP. The clinical characteristics and radiological modalities are summarized in Table 1. Among these affected, 30 (60%) patients were improved by supportive (clinical and endoscopic) management and 20 (40%) were surgically intervened (the interventional outcome (Endoscopic and Surgical management) of these cases will be the future project).

### Genetic Analyses

**Figure 1.**
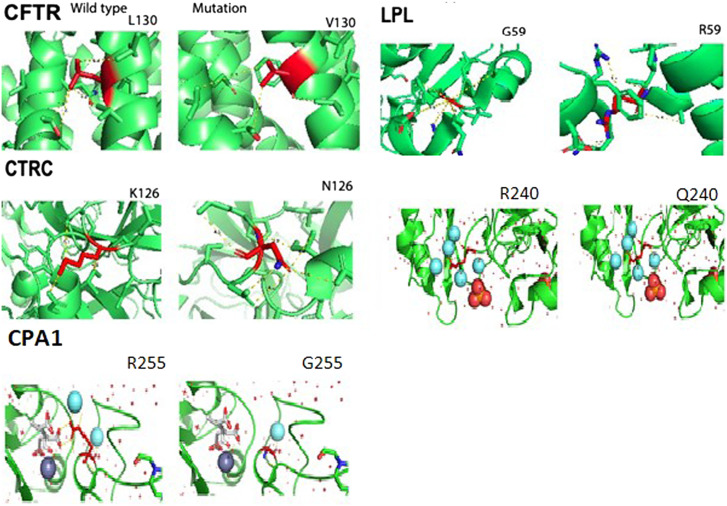
Crystal structure and distance upon mutation are illustrated.

We identified 19 pathogenic disease-causing variants distributed among 9 different genes (4 variants in each *CTRC* and *LPL*; 2 variants in each *PRSS1*, *SPINK1*, *CFTR*, and* CPA1*; and 1 variant in each *CBS, AGL,* and *PHKB*) in a cohort of 50 children. Twenty-three patients (23/50 = 46%) carried at least 1 genetic variant, 16 patients (16/50 = 32%) were compound heterozygous, and trans-heterozygous mutation was observed in 11 patients (11/50 = 22%). The details of variants carriage are shown in Figure 1 and Table 2.

Ten novel variants including [c.378C>T(p.Lys126Asn) and c.719G>A(p.Arg240Gln) in *CTRC*, c.586-3C>A and c.763A>G(p.Arg255Gly) in *CPA1*, c.1160_1161insT(p.Lys387Asnfs*26), c.784C>T(p.Gln262*), c.1139+1G>A, c.175G>A(p.Gly59Arg) in *LPL*, c.388C>G(p.leu130val) in *CFTR*, and c.2327G>A(p.Arg776His in *PHKB*)] were found with a low rare allele frequency in normal population (Table 2).

### In-Silico Protein Analysis of Novel Mutations

Protein simulation and annotations were performed using 3 PyMol was used to physically show the interactions and difference of amino acids in wild and mutant form, as shown in Figure 1, and signifies the changes in interaction with the neighboring amino acids. The △△G energy was calculated to determine whether the protein structure is rendered unstable due to the mutation. Overall stability is calculated from atom potentials and torsion angle potentials. In case of unfavorable torsion angles, the atom potentials may have a higher impact on stability, which results in a stabilizing mutation (Tables 3 and 4).

We also measured the torsion angle changes, the solvent accessibility (%), and depth (Å). The c.378G>T(p.Lys126Asn) variation which did not reduce the stability of protein and this patient (PT 14) also has other mutations which are dominant and explain the single mutation affect.

We found 2 types of heterozygous pathogenic *PRSS1* variants in 12 pediatric patients, including c.365G>A(p.Arg122His) (n = 10), and copy number variable (n = 2). Among patients with CNV (n = 1), *CFTR* c.388C>G(p.Leu130Val) (n = 1) was also detected. We found 2 types of heterozygous pathogenic *SPINK1* variants in 28 patients, including c.101A>G(p.Asn34Ser) (n = 28) and 56-37T>C (n = 22). Among 22 patients with *SPINK1*c.101A>G(p.Asn34Ser) and 56-37T>C variation in other genes, *CBS*;c.434C>T(p.Pro145Leu) (n = 2), *CFTR*;c.2991G>C(p.Leu997Phe) (n = 1), *CTRC*;c.217G>A(p.Ala73Thr) (n = 1), *CTRC*;c.378G>T(p.Lys126Asn) (n = 1), and *CTRC*;c.703G>A(p.Val235Ile) (n = 2) were also detected. Four types of heterozygous pathogenic *CTRC* variants were recognized in 6 patients, including c.378G>T(p.Lys126Asn) (n = 1), c.703G>A(p.Val235Ile) (n = 3), c.217G>A(p.Ala73Thr) (n = 1), and c.719G>A(p.Arg240Gln) (n = 1). Among patients with c.703G>A(p.Val235Ile) (n = 1), *CPA1* c.586-3C>A (n = 1) was also detected. Two patients harboring heterozygous pathogenic *CPA1* variants c.586-3C>A (n = 1), and c.763A>G(p.Arg255Gly) (n = 1) were identified. Among patients with c.763A>G(p.Arg255Gly) (n = 1) *CFTR* c.2991G>C(p.Leu997Phe) (n = 1) was also detected. We found 4 types of heterozygous pathogenic *LPL* variants in 4 patients, including c.1160_1161insT (n = 2), c.784C>T(p.Gln262*) (n = 1), c.1139+1G>A (n = 1), and c.175G>A(p.) (n = 1). Patient with c.175G>A(p.Gly59Arg) also had *PHKB* c.2327G>A(p.Arg776His) (n = 1). Two types of heterozygous pathogenic *CFTR* variants were identified in 5 patients, including c.2991G>C(p.Leu997Phe) (n = 4) and c.388C>G(p.Leu130Val) (n = 1). The c.101A>G(p.Asn34Ser) pathogenic variant in *SPINK1* was the most common variant, followed by c.56-3T>C in *SPINK1* and c.365G>A(p.Arg122His) pathogenic variant in *PRSS1*. They exhibit high penetrance and follow the autosomal dominant pattern of pancreatitis. There was no genotype–phenotype correlation or its effect on the age of symptom onset, among our cohort.

## Discussion

The HCP is one of the genetically determined conditions with different modes of inheritance seen in the pediatric age group. It is also a common cause of chronic pancreatitis in children, followed by hepatobiliary and idiopathic causes.^9^ It results from an imbalance of proteases and their inhibitors in the pancreatic parenchyma thus forming a complex syndrome of varying signs and symptoms including multiple causative pathways that converge into similar phenotypic features.^10^ The incidence of hereditary pancreatitis is 0.3 to 0.5/100 000 cases in the West; however, due to a lack of epidemiological studies, the exact prevalence in the Pakistani population is not known. In this hospital-based study, 64.1% of children had been genetically identified positive among the clinically diagnosed chronic pancreatitis cohort. The gender predilection is the same in pediatric patients though males are more affected in adult literature because of alcohol consumption.^11^ In our study, females are affected more than males. Racial difference does exist in this morbid condition, and Black ethnic origin outnumbers the white population in the West. ^12^

Hereditary chronic pancreatitis affects children at an early age leading to different manifestations later.^13,14^ In our study, there is a delay in diagnosis of about 3.5 years before the confirmation of a diagnosis of HCP could be made. The clinical spectrum of presentation of HCP is no different from chronic pancreatitis of other etiologies.^2,13,15^ Abdominal pain, vomiting, failure to thrive, exocrine insufficiency, and endocrine issues are the main presenting features in the international literature and are similar to our study, as shown in Table 1.^4,16^ The radiological finding of chronic pancreatitis is the primary lead in addition to the clinical background to further investigations in these children. Common radiological findings are calcification in pancreatic parenchyma and dilated and beaded pancreatic duct on ultrasound, CT, or MRCP.^5^

There are a number of mutations identified in recent years in HCP. The majority of them are inherited as an autosomal dominant pattern.^4,17^ Till date 1908 mutations in *CFTR* have been reported in HGMD (http://www.hgmd.cf.ac.uk/ac/index.php) followed by variations in *AGL* (258), *LPL* (244), *CBS* (216), *PRSS1* (69), *SPINK1* (58), *CTRC* (52), *CPAI* (48), and *PHKB* (26). *SPINK1*, *PRSS1*, and *CTRC* variants were over-represented in our population. 

Gene testing of 78 pediatric patients was performed, out of which 50 (50/78, 64%) patients belonging to 44 families were genetically confirmed for pancreatitis. To the best of our knowledge, this is the first study to determine the spectrum and frequency of hereditary pancreatitis in the Pakistani population. We found 19 disease-causing variants in 50 children, out of which 10 were novel. Twenty-three patients (23/50, 46%) carried at least 1 genetic variant, 16 patients (16/50 = 32%) were compound heterozygous, and trans-heterozygous mutations were observed in 11 patients (11/50 = 22%). Children with digenic variations showed faster progression to chronic pancreatitis as compared to variations in a single gene.^17^

SPINK1 is a potent anti-protease that functions as a major activator of intra pancreatic trypsin. *SPINK1* pathogenic variants act as a risk modifier in recurrent acute pancreatitis, thereby lowering the threshold for developing chronic pancreatitis induced by other genetic or environmental factors. In our cohort, *SPINK1* c.101A>G (p.Asn34Ser)-harboring genotype was found as heterozygote, compound heterozygote, and trans-heterozygote in 28 (28/50 = 56%) patients. Most of the patients (22/28 = 79%) harboring c.101A>G (p.Asn34Ser) pathogenic variant also have c.56-37T>C pathogenic variants in *SPINK1*. As shown in literature, c.101A>G (p.Asn34Ser) is in complete linkage with c.56-37T>C, indicating that c.101A>G (p.Asn34Ser) is an ancient evolutionary mutation which arose a long time ago.^18^ As *SPINK1* has autosomal dominant, autosomal recessive, or complex genetic inheritance with co-mutation with another one,^16-18^ in our patients, *SPINK1* is also present in combination with c.378G>T (p.Lys126Asn), c.703G>A (p.Val235Ile), or 217G>A (p.Ala73Thr) pathogenic variants of *CTRC* or c.434C>T (p.Pro145Leu) variant of *CBS*, or c.388C>G (p.Leu130Val) variant of *CFTR*. Compound heterozygotes for *SPINK1* variants were most commonly found in 15 (15/50 = 30%) children. Trans-heterozygotes were also accumulated in patients, and 7 (7/50 = 14%) of our patients carried variants in at least 2 genes. No phenotypic differences between heterozygous and compound heterozygote c.101A>G (p.Asn34Ser) patients were detected. Trans-heterozygotes demonstrate that the carrier of different variants in different pancreatitis-associated genes substantially increases the pancreatitis risk and may in some individuals explain why the disease developed.^18^

Two patients had duplication in *PRSS1* gene; in 1 patient, there is a gain of 1 copy, encompassing exons 1-5 while in the other, there is a gain of 2 copies, encompassing the whole *PRSS1* gene. Gain-of-function pathogenic *PRSS1* variants, including pathogenic missense mutations and pathogenic CNVs such as gene duplication or triplication, have already been reported as the most common PRSS1 disorder.^19^ Schnúr et al^20^ reported that c.410C>T (p.Thr137Met), c.508A>G (p.Lys170Glu), and c.623G>C (p.Gly208Ala) variants in *PRSS1*are rare and predominant in subjects of Asian origin. None of our patients had these variants rather they had the most commonly “classic pathogenic variant” c.365G>A (p.Arg122His).

Four children with HCP, had unusual mutation identified as lipoprotein lipase deficiency (*LPL*), c.1160_1161insT, and c.175G>A(p.Gly59Arg) variants had autosomal recessive pattern while c.784C>T(p.Gln262*), and c.1139+1G>A had autosomal dominant pattern which is similar to international literature.^21^ c.175G>A(p.Gly59Arg) variant was found in combination of c.2327G>A(p.Arg776His) variant in *PHKB1.* They were being managed as hypertriglyceridemia-induced chronic pancreatitis. Among these 4 children, 2 were sisters, and the other 2 belonged to different families. 

Chronic pancreatitis caused by *CFTR* is inherited in an autosomal recessive or complex manner due to compound heterozygosity for *CFTR* or a combination of defects in *CFTR* and other genes.^22^ Cationic trypsinogen mutation (*PRSS1*) generally does not require added risk factors, but others like *SPINK1* and *CFTR* are considered disease modifiers and require additional factors for manifestation.^23^ The risk factors identified in these children are abdominal trauma, hypertriglyceridemia, and familial pattern. In addition to these risk factors, the trans-heterozygous mutation is also considered a risk factor like *CFTR* and *SPINK1* co-mutation.^18,23^ Diagnosing early chronic pancreatitis remains challenging in pediatrics as in adults. Previous studies reported a 30%-73% association of genetic mutations with pancreatitis.^24^ The mode of presentation may be unusual in children owing to the presence of unique genetic mutations.

Hereditary chronic pancreatitis accounts for approximately 20%-25% of all cases in childhood. About 4 probands had no family history of chronic pancreatitis, although the mutation was inherited in all cases by at least 1 parent, indicating a low penetrance of these mutations. This finding is in contrast to the high penetrance reported for the c.365G>A(p.Arg122His) mutations in *PRSS1*. Thus, trypsinogen mutations display considerable variability with respect to penetrance. Although c.365G>A(p.Arg122His) mutation has also been in “sporadic” cases of chronic pancreatitis without a family history. The high penetrance reported may reflect more the penetrance in certain chronic pancreatitis families (sharing a similar genetic background) than the penetrance of these mutations in general.^25^

Medical management is the cornerstone of treatment in this highly morbid condition. Most patients are managed with dietary restrictions, analgesics, pancreatic enzyme replacement therapy, and proton pump inhibitors in addition to insulin for endocrine complications and are similar to the current study. Endoscopic retrograde cholangiopancreatography has a therapeutic role in pancreatitis with stone impaction/obstruction or sometimes needed for papillotomy/sphincterotomy, similarly, 5 patients in this study required endoscopic retrograde cholangiopancreatography (ERCP), 2 had stent placement, and 3 with papillotomy/sphincterotomy.^26^ For relentless pain and discomfort and with failure of medical management, surgical intervention in the form of pancreatectomy or islet cell transplantation is required to alleviate the symptoms and improve the quality of life.^26-28^ The majority of our children had a good quality of life on medical measures, but 20% of children required surgical interventions in the form of cyst gastrostomy and Puestow procedure for relief from pseudocyst and pain, respectively. 

The novel mutations mapped in *LPL*, *CFTR*, *CTRC*, and *PHKB* genes show a potential for new finding in pancreatitis and their clinical-genetic importance for diagnostic and pharmaceutics. Although this study had a small sample size, it includes an expanded panel of genes in case of hereditary pancreatitis. This is a very important finding in the Pakistani cohort for further clinical-genetic criteria revision at the local level. 

Limitation of our study includes the retrospective nature, single-center database, and potential missing of data could not be ruled out; however, an attempt is made to share the perspective of hereditary chronic pancreatitis in Pakistani children. We still need further cellular and drug testing studies to evaluate these new novel mutations.

## Conclusion

This is the first-ever long clinical and genetic follow-up study on chronic pancreatitis disease in Pakistani children. Heterogeneous genetic factors play a significant role in disease manifestation. Genetic variants confer susceptibility to HCP, and which polymorphisms modify the prognosis and treatment response that leads to chronic pancreatitis will also be important in determining the timing of genetic testing and subsequent intervention. Micromanagement with painkillers, dietary modifications and exocrine/endocrine support keeps a good quality of life, but surgical intervention is definitely needed if medical measures fail. It shows genetic and clinical diagnosis, and follow-up is critical for accurate and personalized medication. Genetic testing in the future will therefore require the inclusion of many polymorphisms, and the results will require careful interpretation. It is conceivable that therapy will be targeted at preventing HCP through genetic predisposition.

## Figures and Tables

**Supplementary Figure 1.**
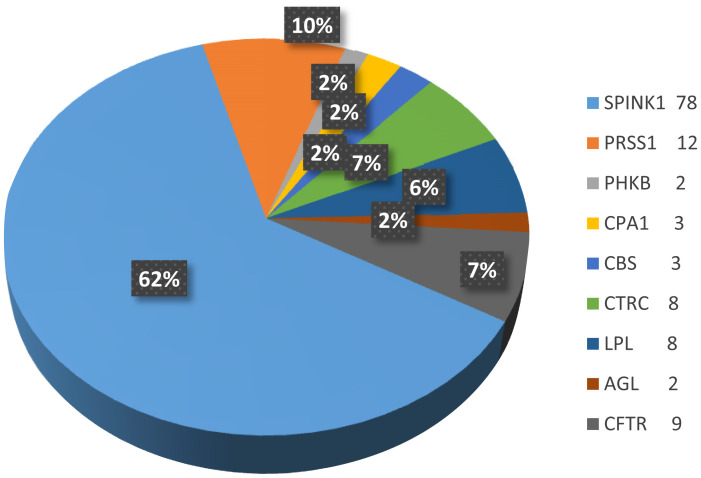
The allele count of corresponding genes.

**Table 1.** Demographic, Risk Factors, Clinical Presentation, and Radiological Modalities in 50 Pakistani Patients with Hereditary Pancreatitis

Characteristics	Number (%)
Demographic	
Mean age at presentation (mean ± SD in years)	6.81 ± 2.3
Mean age at onset of symptoms (mean ± SD in years)	3.3 ± 1.2
Females	31 (62%)
Males	19 (38%)
Consanguinity	34 (68%)
Risk factors	
Positive family history	25 (50%)
Hypertriglyceridemia	11 (22%)
Abdominal trauma	4 (8%)
Hypercalcemia	2 (4%)
Clinical presentation	
Abdominal pain	49 (98%)
Vomiting	45 (90%)
Failure to thrive	34 (68%)
Fever	19 (38%)
Ascites	7 (14%)
Jaundice	4 (8%)
Steatorrhea	3 (6%)
Diabetes mellitus	3 (6%)
Imaging findings	
Pancreatic duct stenosis or dilation	37 (74%)
Pancreatic duct stones	3 (6%)
Pancreatic duct pseudocyst	7 (14%)
Pancreatic duct calcification	9 (18%)

**Table 2.** Genetic Data of All Mutations of Known and Candidate Genes Found in our Cohort

Family ID/Consanguinity	Patient ID	Gender	Gene	Exon/Intron	cDNA	Amino Acid	Zygosity	gnomAD (Homozyote/Allele Count/Allele Number)	Frequency on gnomAD	Mutation Taster	Polyphen
F 1/yes	Pt 1	Female	*PRSS1*	Exon 3	c.365G>A	p.Arg122His	Het	0/3/251304	0.0000119	DC	B.001
	Pt 2	Female	*PRSS1*	Exon 3	c.365G>A	p.Arg122His	Het	0/3/251304	0.0000119	DC	B.001
	Pt 3	Female	*PRSS1*	Exon 3	c.365G>A	p.Arg122His	Het	0/3/251304	0.0000119	DC	B.001
F 2/yes	Pt 4	Female	*SPINK1*	Exon 3	c.101A>G	p.Asn34Ser	Hom	23/2537/281004	0.00903	P	B.005
			*SPINK1*	Intron 1	c.56-37T>C	NA	Hom	21/2317/280614	0.00826	-	-
F 3/yes	Pt 5	Male	*PRSS1*	Exon 3	c.365G>A	p.Arg122His	Het	0/3/251304	0.0000119	DC	B.001
F 4/No	Pt 6	Female	*CTRC*	Exon 7	c.719G>A	p.Arg240Gln	Het	0/7/251258	0.0000318	P	B.012
F 5/yes	Pt 7	Male	*SPINK1*	Exon 3	c.101A>G	p.Asn34Ser	Het	23/2537/281004	0.00903	P	B.005
			*SPINK1*	Intron 1	c.56-37T>C	NA	Het	21/2317/280614	0.00826	-	-
F 6/ Yes	Pt 8	Male	*SPINK1*	Exon 3	C.101A>G	p.Asn34Ser	Hom	23/2537/281004	0.00903	P	B.005
F 7/yes	Pt 9	Female	*PRSS1*	Exon 3	c.365G>A	p.Arg122His	Het	0/3/251304	0.0000119	DC	B.001
F 8/yes	Pt 10	Female	*SPINK1*	Exon 3	c.101A>G	p.Asn34Ser	Hom	23/2537/281004	0.00903	P	B.005
F 9/	Pt 11	Female	*SPINK1*	Exon 3	c.101A>G	p.Asn34Ser	Het	23/2537/281004	0.00903	P	B.005
F 10/yes	Pt 12	Female	*PRSS1*		MLPA	-	Het	-	0	-	-
F 11/No	Pt 13	Female	*SPINK1*	Exon 3	c.101A>G	p.Asn34Ser	Het	23/2537/281004	0.00903	P	B.005
			*SPINK1*	Intron 1	c.56-37T>C	NA	Het	21/2317/280614	0.00826	-	NA
F 12/No	Pt 14	Male	*SPINK1*	Exon 3	c.101A>G	p.Asn34Ser	Het	23/2537/281004	0.00903	P	B.005
			*SPINK1*	Intron 1	c.56-37T>C	NA	Het	21/2317/280614	0.00826	-	-
			*CTRC*	Exon 5	c.378G>T	p.Lys126Asn	Hom	0/1/251488	0.00000398	DC	D.99
F 13/yes	Pt 15	Female	*CPA1*	Exon 5	c.586-3C>A	NA	Het	3/213/282562	0.000754	-	-
			*CTRC*	Exon 7	c.703G>A	p.Val235Ile	Het	3/293/282822	0.00104	DC	D.969
F 14/yes	Pt 16	Female	*SPINK1*	Exon 3	c.101A>G	p.Asn34Ser	Hom	23/2537/281004	0.00903	P	B.005
	Pt 17	Female	*SPINK1*	Exon 3	c.101A>G	p.Asn34Ser	Hom	23/2537/281004	0.00903	P	B.005
F 15/yes	Pt 18	Female	*PRSS1*	Exon 3	c.365G>A	p.Arg122His	Het	0/3/251304	0.0000119	DC	B.001
	Pt 19	Female	*PRSS1*	Exon 3	c.365G>A	p.Arg122His	Het	0/3/251304	0.0000119	DC	B.001
F 16/yes	Pt 20	Female	*SPINK1*	Exon 3	c.101A>G	p.Asn34Ser	Het	23/2537/281004	0.00903	P	B.005
F 17/No	Pt 21	Male	*SPINK1*	Exon 3	c.101A>G	p.Asn34Ser	Het	23/2537/281004	0.00903	P	B.005
			*SPINK1*	Intron 1	c.56-37T>C	NA	Het	21/2317/280614	0.00826	-	NA
F 18/Yes	Pt 22	Male	*SPINK1*	Exon 3	c.101A>G	p.Asn34Ser	Hom	23/2537/281004	0.00903	P	B.005
			*SPINK1*	Intron 1	c.56-37T>C	NA	Hom	21/2317/280614	0.00826	-	-
			*CTRC*	Exon 7	c.703G>A	p.Val235Ile	Het	3/293/282822	0.00104	DC	D.969
F 19/Yes	Pt 23	Female	*CFTR*	Exon 19	2991G>C	p.Leu997Phe	Hom	3/627/282204	0.00222	DC	D.99
			*CPA1*	Exon 7	c.763A>G	p.Arg255Gly	Hom	0/12/251432	0.0000477	P	
F 20/Yes	Pt 24	Female	*SPINK1*	Exon 3	c.101A>G	p.Asn34Ser	Hom	23/2537/281004	0.00903	P	B.005
			*SPINK1*	Intron 1	c.56-37T>C	NA	Hom	21/2317/280614	0.00826	-	-
			*CTRC*	Exon 3	c.217G>A	p.Ala73Thr	Hom	1/167/251050	0.00066	DC	D .99
F 21/No	Pt 25	Female	*SPINK1*	Exon 3	c.101A>G	p.Asn34Ser	Het	23/2537/281004	0.00903	P	B.005
			*SPINK1*	Intron 1	c.56-37T>C	NA	Het	21/2317/280614	0.00826	-	-
F 22/Yes	Pt 26	Female	*SPINK1*	Exon 3	c.101A>G	p.Asn34Ser	Hom	23/2537/281004	0.00903	P	B.005
			*SPINK1*	Intron 1	c.56-37T>C	NA	Hom	21/2317/280614	0.00826	-	-
F 23/Yes	Pt 27	Male	*SPINK1*	Exon 3	c.101A>G	p.Asn34Ser	Het	23/2537/281004	0.00903	P	B.005
			*SPINK1*	Intron 1	c.56-37T>C	NA	Het	21/2317/280614	0.00826	-	-
			*CBS*	Exon 5	c.434C>T	p.Pro145Leu	Hom	0/1/249936	0.000004	DC	D 1
F 24/No	Pt 28	Female	*SPINK1*	Exon 3	c.101A>G	p.Asn34Ser	Het	23/2537/281004	0.00903	P	B.005
			*SPINK1*	Intron 1	c.56-37T>C	NA	Het	21/2317/280614	0.00826	-	-
			*CTRC*	Exon 7	c.703G>A	p.Val235Ile	Het	3/293/282822	0.00104	DC	D.969
F 25/Yes	Pt 29	Female	*LPL*	Exon 6	c.1160_1161insT	p.Lys387Asnfs*26	Hom	-	-	DC	-
	Pt 30	Female	*LPL*	Exon 6	c.1160_1161insT	p.Lys387Asnfs*26	Hom	-	-	DC	-
F 26/Yes	Pt 31	Male	*LPL*	Exon 6	c.784C>T	p.Gln262*	Het	0/1/251224	0.00000398	DC	
			*LPL*	Intron 7	c.1139+1G>A	NA	Het	0/2/251110	0.00000796	-	-
F 27/Yes	Pt 32	Male	*AGL*	Exon 6	c.753_756del	p.Asp251Glufs*23	Hom	0/9/282744	0.000032	DC	-
F 28/Yes	Pt 33	Male	*SPINK1*	Exon 3	c.101A>G	p.Asn34Ser	Hom	23/2537/281004	0.00903	P	B.005
			*SPINK1*	Intron 1	c.56-37T>C	NA	Hom	21/2317/280614	0.00826	-	-
F 29/Yes	Pt 34	Female	*SPINK1*	Exon 3	c.101A>G	p.Asn34Ser	Het	23/2537/281004	0.00903	P	B.005
			*SPINK1*	Intron 1	c.56-37T>C	NA	Het	21/2317/280614	0.00826	-	-
			*CFTR*	Exon 19	c.2991G>C	p.Leu997Phe	Hom	3/627/282204	0.00222	DC	D.99
F 30/Yes	Pt 35	Female	*PRSS1*		MLPA	-	Het		-		
			*CFTR*	Exon 4	c.388C>G	p.Leu130Val	Het	0/11/250878	0.000044	DC	D .99
F 31/Yes	Pt 36	Female	*SPINK1*	Exon 3	c.101A>G	p.Asn34Ser	Hom	23/2537/281004	0.00903	P	B.005
			*SPINK1*	Intron 1	c.56-37T>C	NA	Hom	21/2317/280614	0.00826	-	-
F 32/Yes	Pt 37	Male	*SPINK1*	Exon 3	c.101A>G	p.Asn34Ser	Het	23/2537/281004	0.00903	P	B.005
			*SPINK1*	Intron 1	c.56-37T>C	NA	Het	21/2317/280614	0.00826	-	-
F 33/No	Pt 38	Female	*LPL*	Exon 2	c.175G>A	p.Gly59Arg	Hom	0/7/251484	0.0000278	DC	D .978
			*PHKB*	Exon 26	c.2327G>A	p.Arg776His	Hom	0/8/282092	0.0000284	DC	D .999
F 34/Yes	Pt 39	Female	*SPINK1*	Exon 3	c.101A>G	p.Asn34Ser	Hom	23/2537/281004	0.00903	P	B.005
			*SPINK1*	Intron 1	c.56-37T>C	NA	Hom	21/2317/280614	0.00826	-	-
F 35/No	Pt 40	Female	*SPINK1*	Exon 3	c.101A>G	p.Asn34Ser	Hom	23/2537/281004	0.00903	P	B.005
			*SPINK1*	Intron 1	c.56-37T>C	NA	Hom	21/2317/280614	0.00826	-	-
F 36/No	Pt 41	Female	*SPINK1*	Exon 3	c.101A>G	p.Asn34Ser	Hom	23/2537/281004	0.00903	P	B.005
			*SPINK1*	Intron 1	c.56-37T>C	NA	Hom	21/2317/280614	0.00826	-	-
F 37/Yes	Pt 42	Female	*CFTR*	Exon 19	c.2991G>C	p.Leu997Phe	Hom	3/627/282204	0.00222	DC	D.99
	Pt 43	Female	*CFTR*	Exon 19	c.2991G>C	p.Leu997Phe	Hom	3/627/282204	0.00222	DC	D.99
F 38/Yes	Pt 44	Female	*PRSS1*	Exon 3	c.365G>C	p.Arg122His	Het	0/3/251304	0.0000119	DC	B.001
F 39//Yes	Pt 45	Male	*SPINK1*	Exon 3	c.101A>G	p.Asn34Ser	Het	23/2537/281004	0.00903	P	B.005
			*SPINK1*	Intron 1	c.56-37T>C	NA	het	21/2317/280614	0.00826	-	-
			*CBS*	Exon 5	c.434C>T	p.Pro145Leu	Het	0/1/249936	0.000004	DC	D1.00
F 40/Yes	Pt 46	Female	*PRSS1*	Exon 3	c.365G>A	p.Arg122His	Het	0/3/251304	0.0000119	DC	B.001
F 41/Yes	Pt 47	Male	*PRSS1*	Exon 3	c.365G>A	p.Arg122His	Het	0/3/251304	0.0000119	DC	B.001
F 42/Yes	Pt 48	Female	*SPINK1*	Exon 3	c.101A>G	p.Asn34Ser	Hom	23/2537/281004	0.00903	P	B.005
			*SPINK1*	Intron 1	c.56-37T>C	NA	Hom	21/2317/280614	0.00826	-	-
F 43/No	Pt 49	Male	*SPINK1*	Exon 3	c.101A>G	p.Asn34Ser	Hom	23/2537/281004	0.00903	P	B.005
			*SPINK1*	Intron 1	c.56-37T>C	NA	Hom	21/2317/280614	0.00826	-	-
F 44/No	Pt 50	Male	*SPINK1*	Exon 3	c.101A>G	p.Asn34Ser	Hom	23/2537/281004	0.00903	P	B.005
			*SPINK1*	Intron 1	c.56-37T>C	NA	Hom	21/2317/280614	0.00826	-	-

B, benign; D, damaging protein; DC, disease causing; gnomAD, genome aggregation database; Het, heterozygous; Hom, homozygous; P, polymorphism.

**Table 3.** Novel Mutation △△G and Torsion Angle Measurement

Gene	PDB No	Mutations	(△△G)	Stability of Protein	Torsion Angle	Zygosity
*CFTR*	5UAK	Leu130Val	−0.67	Reduced stability	Unfavorable	Het
*CTRC*	4H4F	Lys126Asn	0.56	Increased stability	Unfavorable	Het
		Arg240Gln	−6.790	Reduced stability	Favorable	Het
*CPA1*	2V77	Arg255Gly	−3.5	Reduced stability	Unfavorable	Hom
*LPL*	60AZ,6E7K	Gly59Arg	−3.12	Reduced stability	Unfavorable	Hom

Het, heterozygous; Hom, homozygous.

**Table 4.** Solvent Accessibility and Depth of Novel Mutations

Type	Secondary Structure	Solvent Accessibility (%)	Depth (Å)
*CFTR*: Leu130Val			
Wild type	H	33.8	4.1
Mutant	H	35	4.2
*CTRC*: Lys126Asn			
Wild type	P	54.8	3.5
Mutant	P	65.2	3.5
*CTRC*: Arg240Gln			
Wild type	A	66.2%	3.5
Mutant	A	65.1%	3.6
*CPA1*: Arg255Gly			
Wild type	H	3.6%	6.0
Mutant	A	11.0%	9.1
*LPL*:Gly59Arg			
Wild type	G	1.1	5.5
Mutant	G	20.2	4.7

**Supplementary Table 1:** The Demographic, Clinical Characteristics with Brief Management Summary

**Family ID**	**Pt. ID **	**Age**	**Sex**	**Age at Present (Years)**	**Duration**	**F/H**	**Triglycerides**	**Ca levels**	**Amylase**	**Lipase**	**USG Abd**	**MRCP**	**Consanguinity**	**Exocrine Insufficiency**	**TX**	**Mutation**
**F1**	Pt 1	14	Female	9	5	Yes	170	8.9	84	34	2	4	Yes	No	PUESTOW	PRSS1
	Pt 2	8	Female	7	1	Yes	163	8.7	162	75	2,4	4	Yes	No	PUESTOW	PRSS1
	Pt 3	11	Female	10	1	Yes	122	9.2	123	231	3	4	Yes	No	PUESTOW	PRSS1
**F2**	Pt 4	3	Female	3.5	0.5	No	165	9.5	187	230	2	2	Yes	No	SUPPORTIVE	SPINK1
**F3**	Pt 5	4	Male	3.5	0.5	Yes	120	8.9	123	213	3	3	Yes	No	SUPPORTIVE	PRSS1
**F4**	Pt 6	8	Female	6	2	No	91	8.4	83	97	2	3	No	Yes	SUPPORTIVE	CTRC
**F5**	Pt 7	6	Male	4	2	Yes	213	8.4	562	564	2+4	4	Yes	No	SUPPORTIVE	SPINK1
**F6**	Pt 8	13	Male	9	4	No	222	9.1	522	290	3	3	No	No	SUPPORTIVE	SPINK1
**F7**	Pt 9	9	Female	8	1	Yes	150	9.4	161	184	2	3	No	No	SUPPORTIVE	PRSS1
**F8**	Pt 10	11	Female	6	5	No	210	9.2	499	333	3	3	Yes	No	SUPPORTIVE	SPINK1
**F9**	Pt 11	7	Female	6	1	No	240	9.1	323	159	3	3,4	Yes	No	SUPPORTIVE	SPINK1
**F10**	Pt 12	7	Female	6	2	No	65	9.1	498	234	2+4	5	Yes	Yes	SUPPORTIVE	PRSS1
**F11**	Pt 13	10	Female	9.5	0.5	Yes	178	8.6	558	1153	2	3	No	No	SUPPORTIVE	SPINK1
**F12**	Pt 14	11	Male	7	4	Yes	78	7.5	179	320	2,4	3	No	No	SUPPORTIVE	SPINK1, CTRC
**F13**	Pt 15	10	Female	8	2	No	89	10.6	75	83	3	3	Yes	No	SUPPORTIVE	CTRC, CPA1
**F14**	Pt 16	12	Female	8	4	Yes	165	8.9	116	45	4	5	Yes	No	CYSTOGASTROSTOMY	SPINK 1
	Pt 17	12	Female	11.5	0.5	Yes	170	8.8	320	340	4	5	Yes	No	CYSTOGASTROSTOMY	SPINK 1
**F15**	Pt 18	11	Female	7	4	Yes	123	9.1	111	67	2	3	Yes	Yes	CYSTOGASTROSTOMY	PRSS1
	Pt 19	9	Female	7	2	Yes	154	8.9	94	87	2	3	Yes	Yes	SUPPORTIVE	PRSS1
**F16**	Pt 20	6.5	Female	4.5	2	No	167	9.5	132	776	2	3	Yes	No	SUPPORTIVE	SPINK1
**F17**	Pt 21	16	Male	10	6	No	115	9.2	75	89	2	2	Yes	No	SUPPORTIVE	SPINK 1
**F18**	Pt 22	6	Male	4	2	No	145	9.7	302	625	3	3	Yes	No	SUPPORTIVE	SPINK1, CTRC
**F19**	Pt 23	8	Female	6	2	Yes	187	8.9	230	341	2	3	Yes	No	SUPPORTIVE	CFTR, CPA1
**F20**	Pt 24	9	Female	7	2	No	137	10.7	33	167	2	3	Yes	No	SUPPORTIVE	SPINK1, CTRC
**F21**	Pt 25	11	Female	9	2	No	109	8.7	2280	74	2	3	No	Yes	SUPPORTIVE	SPINK 1
**F22**	Pt 26	15	Female	11	4	No	64	9.5	1097	890	1	1	Yes	No	SUPPORTIVE	SPINK 1
**F23**	Pt 27	9	Male	7	2	No	115	7.9	317	192	2,4	3,5	Yes	No	SUPPORTIVE	SPINK, CBS
**F24**	Pt 28	8	Female	4	4	No	165	9.1	394	293	3	3,5	No	No	SUPPORTIVE	SPINK1, CTRC
**F25**	Pt 29	6	Female	5.9	0.1	Yes	1555	8	151	230	1	1	Yes	No	TX HYPERTRIG	LPL
	Pt 30	12	Female	8	4	Yes	1480	7.2	21	23	2	3	Yes	No	SUPPORTIVE	LPL
**F26**	Pt 31	8	Male	5	3	No	980	9.2	422	40	2	3	No	No	SUPPORTIVE	LPL
**F27**	Pt 32	7.5	Male	5	2	No	343	8.4	233	799	2	3	Yes	No	SUPPORTIVE	AGL
**F28**	Pt 33	10	Male	7	3	No	388	9.4	596	376	2	3	Yes	No	SUPPORTIVE	SPINK 1
**F29**	Pt 34	10	Female	6	4	No	122	9.1	71	49	2	3	Yes	No	SUPPORTIVE	SPINK 1, CFTR
**F30**	Pt 35	10	Female	8	2	No	288	9.9	311	798	2	3	Yes	No	SUPPORTIVE	PRSS1, CFTR
**F31**	Pt 36	13	Female	11	2	No	177	8.8	1122	3300	2	3	No	Yes	SUPPORTIVE	SPINK 1
**F32**	Pt 37	11	Male	8	3	No	211	8.9	68	41	2	3	Yes	No	SUPPORTIVE	SPINK 1
**F33**	Pt 38	6	Female	4	2	No	622	9.1	499	1090	3	3,4	Yes	Yes	SUPPORTIVE	LPL, PHKB
**F34**	Pt 39	12	Female	8	4	No	215	9.2	93	60	2	2	No	No	SUPPORTIVE	SPINK 1
**F35**	Pt 40	8	Male	8	0.1	No	56	7.8	1093	2971	2	2	No	No	SUPPORTIVE	SPINK 1
**F36**	Pt 41	3	Female	2	1	No	44	8.8	1540	5273	1	1	Yes	No	SUPPORTIVE	SPINK 1
**F37**	Pt 42	15	Male	12	3	Yes	143	8.7	98	86	3,4	2	Yes	Yes	ON INSULINE, CREON	CFTR
	Pt 43	12	Male	9	3	Yes	99	9.1	506	11	3	3	Yes	No	SUPPORTIVE	CFTR
**F38**	Pt 44	6	Female	5	1	Yes	167	8.9	345	541	2	3	Yes	No	SUPPORTIVE	PRSSI
**F39**	Pt 45	5	Male	4	1	Yes	167	8.9	340	340	2	4	Yes	No	SUPPORTIVE	SPINK1, CBS
**F40**	Pt 46	10	Female	6	4	No	170	8.9	62	131	3	4	No	No	SUPPORTIVE	PRSS1
**F41**	Pt 47	6	Male	5.5	0.6	No	132	8.8	412	96	2	2	No	No	SUPPORTIVE	PRSS1
**F42**	Pt 48	10	Male	5	5	No	143	9.1	133	51	3,5	2	No	No	SUPPORTIVE	SPINK1
**F43**	Pt 49	15	Male	10	5	No	129	9.2	933	2200	2	3	Yes	Yes	SUPPORTIVE	SPINK1
**F44**	Pt 50	12	Male	7	5	No	131	9.1	292	411	2	3	Yes	No	SUPPORTIVE	SPINK1
